# Acute Intratonsillar Abscess in an Adult: A Case Report and Literature Review

**DOI:** 10.7759/cureus.51657

**Published:** 2024-01-04

**Authors:** Alexandra F Corbin, Arunima Vijay, Jacob Fried, Michele M Carr

**Affiliations:** 1 Otolaryngology, Jacobs School of Medicine and Biomedical Sciences at the University at Buffalo, Buffalo, USA; 2 Diagnostic Radiology, University of Florida, Gainesville, USA

**Keywords:** incision and drainage of abscess, needle aspiration, antibiotics therapy, ct (computed tomography) imaging, peritonsillar abscess, intratonsillar abscess

## Abstract

Intratonsillar abscess (ITA) is rarely reported. Here, we present an uncommon case of acute ITA in an adult, discuss the evaluation and treatment plan, and review the ITA literature.

The abscess reported in the present study was diagnosed through a combination of clinical findings and computed tomography imaging, and treatment included drainage, intravenous (IV) clindamycin, and IV dexamethasone.

The literature reports 72 ITA cases with specified treatments: 21 (29.2%) in adults, 19 (26.4%) in children, and 32 (44.4%) in patients of unspecified ages. Among them, 25 (34.7%) responded to antibiotics alone, 11 (15.3%) to needle aspiration and antibiotics, and 36 (50.0%) needed further intervention.

Based on the presented case and literature review, we suggest the use of IV antibiotics with needle aspiration as the primary treatment for acute ITA. Incision and drainage (I&D) with antibiotics should be reserved for cases unresponsive to initial measures, and tonsillectomy is recommended for recurrent post-I&D cases.

## Introduction

Acute intratonsillar abscess (ITA) is an uncommon infection that is challenging to identify because its clinical features overlap with those of peritonsillar abscess (PTA). ITA is characterized by an accumulation of pus within the tonsillar parenchyma, whereas PTA consists of pus accumulating deep to the tonsil [1,2].

ITA can occur as a sequela of acute follicular tonsillitis or as a result of bacterial seeding in the tonsils through the bloodstream or lymphatic system [3,4]. However, our current understanding of this pathogenesis is based on fewer than 200 reported cases [5].

Given the rarity of ITA, there is no defined protocol for the most efficient diagnosis and optimal treatment of this infection. This case report presents the use of computed tomography (CT) imaging to diagnose ITA, along with successful management.

This case was previously presented as a meeting poster at the American Academy of Otolaryngology Annual Meeting on October 2, 2023.

## Case presentation

A 32-year-old Hispanic female with no relevant past medical history presented to urgent care with worsening throat pain, progressive dysphagia, trismus, voice changes, and fevers for the past week. She was diagnosed with PTA based on clinical examination and referred to the emergency department (ED).

After assessment in the ED, she was admitted to the hospital and referred to the otolaryngology team. The patient reported fever and chills but no trismus. On otolaryngologic examination, she showed no signs of respiratory distress. However, she had mild cervical adenopathy, a midline uvula with tonsillar asymmetry, and a Brodsky scale 2+ right tonsil with 3+ left tonsil [6]. She had bilateral posterior oropharyngeal erythema, and the left tonsil was actively draining a yellowish-white fluid purulence from the inferior pole, lateral to the tongue base.

**Figure 1:**
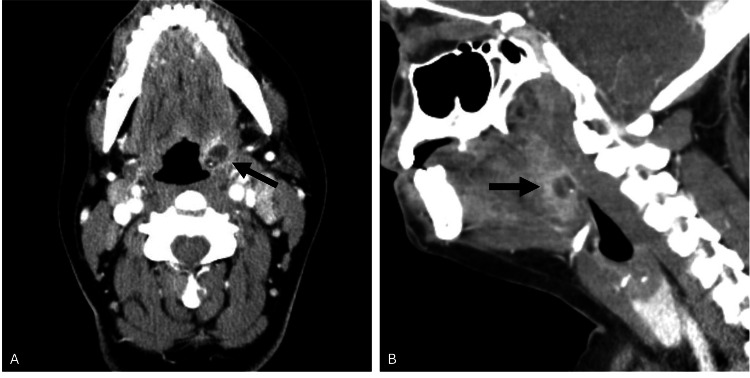
Computed tomography images of the neck Axial (A) and sagittal (B) contrast-enhanced CT scans of the neck demonstrate the presence of an isolated left ITA causing left palatine tonsil enlargement with central hypodensity and rim enhancement (black arrows). Septation is visualized as the hyperdense line within the left palatine tonsil. A small, circular, hyperdense calcification is visible in the inferior-posterior region of the left tonsil. ITA - intratonsillar abscess

Contrast-enhanced cervical CT imaging revealed a 1.5x1.1x1.1 cm left palatine tonsil with a central area of hypoattenuation, surrounded by rim enhancement. A single septation and a 2 mm calcification were also observed within the tonsil of interest. Additionally, there was notable left-sided reactive cervical adenopathy (Figure 1).

Laboratory testing did not show an elevated white blood cell count or any other abnormalities in the complete blood count or comprehensive metabolic panel. An attempt to aspirate the ITA with a needle was unsuccessful. Subsequently, the left tonsil was probed with a curved hemostat, resulting in yellowish-white purulent drainage, pressure relief, and improvement in the patient's voice. No fluid was sent for cultures. IV clindamycin (a single 900 mg dose) and IV dexamethasone (a single 10 mg dose) were initiated.

The patient was discharged on oral clindamycin (300 mg every six hours for 10 days) and oral dexamethasone (4 mg Medrol® Dosepak) after spending approximately four hours in the ED and was scheduled for a one-week outpatient follow-up, at which point her symptoms had completely resolved. No bloodwork was done at this follow-up.

## Discussion

While both ITA and PTA exhibit similar symptoms, previous reports suggest that ITA may lead to a milder clinical presentation. Both infections can present with a sore throat and fever for several days, along with otalgia, trismus, and voice changes. However, ITA is reported to cause less dysphagia, trismus, and voice changes compared to PTA [1,7,8]. Our case is the first to document an ITA presentation with a significant overlap in symptom severity with PTA.

Due to the limited number of reported cases in the literature, there is no consensus regarding the optimal diagnostic method for ITA [4]. We recommend conducting a thorough history and physical examination to screen for ITA, followed by cervical CT imaging and abscess drainage for confirmation. Contrast-enhanced CT is beneficial as it allows for improved examination of the tonsillar tissue surrounding the abscess, which is necessary to distinguish ITA from PTA [1].

In our case, contrast-enhanced CT findings confirmed the intratonsillar location of the infection and, therefore, suggested a diagnosis of ITA despite the ambiguity in the patient's symptoms. Purulent drainage from the abscess ultimately confirmed the diagnosis. The diagnostic method we recommend is primarily applicable to adult populations, as it may be challenging to encourage children to cooperate during cervical CT scans. For pediatric cases, ultrasound emerges as a valuable alternative to CT, building upon its established track record for diagnosing PTA due to its cost-effectiveness and diagnostic accuracy. Ultrasound's user-friendliness, especially when assessing areas like the tonsils, offers a more comfortable and less stressful experience for children compared to CT scans, which often require breath-holding and an extended period of immobility [9-11].

A comprehensive review of PubMed and Google Scholar identified 22 unique articles, reporting a total of 171 ITA cases between April 1991 and July 2023. Among these studies, treatment regimens were described in 19 articles (86.4%), covering 72 cases (42.1%). Out of these cases, 21 (29.2%) occurred in adults, 19 (26.4%) in children, and 32 (44.4%) in patients of unspecified ages. The most common approach to resolving ITA was the use of antibiotics alone (25 cases, 34.7%), followed by needle aspiration with or without antibiotics (11 cases, 15.2%). However, 36 cases (50%) required additional interventions such as incision and drainage (I&D) and/or tonsillectomy, either individually or in combination with needle aspiration and/or antibiotics (Tables 1-3). Currently, there is no standardized treatment protocol for managing ITA.

**Table 1:** Treatment of adult (>18 years old) ITA cases ^a ^Method of antibiotic administration was not reported. NR - not reported; BID - two times a day; ITA - intratonsillar abscess

Author	Year	Age (years)/sex	Resolution treatment method	Antibiotic	Antibiotic dosage/duration (days)
Añaguari et al. [20]	2016	50/F	Tonsillectomy	-	-
Cheong et al. [21]	2015	42/M	Tonsillectomy	-	-
Childs et al. [3]	1991	37/F	Tonsillectomy	-	-
		45/M	Tonsillectomy	-	-
		21/F	Tonsillectomy	-	-
		27/F	Tonsillectomy	-	-
		24/M	Tonsillectomy	-	-
Esmaili et al. [15]	2018	40/M	Needle aspiration, incision and drainage	-	-
Gan et al. [22]	2008	33/M	Unspecified drainage	-	-
Hsu et al. [23]	2008	32/M	IV antibiotics, needle aspiration	ampicillin sulbactam	NR/NR
Mohammed et al. [24]	2017	42/M	IV antibiotics, needle aspiration, tonsillectomy	benzylpenicillin metronidazole	NR/NR
Ng et al. [25]	2018	19/F	IV antibiotics, needle aspiration, incision and drainage	NR	NR/NR
Rai et al. [16]	2020	35/F	IV antibiotics, needle aspiration	amoxicillin	1 g/NR
				clavulanic acid	200 mg every 12 hours/NR
				metronidazole	100 ml every eight hours/NR
		34/M	Oral antibiotics, needle aspiration	cefpodoxime	200 mg/NR
				clavulanic acid	125 mg BID/NR
		30/F	IV antibiotics, needle aspiration	amoxicillin	1 g/NR
				clavulanic acid	200 mg every 12 hours/NR
Singh et al. [5]	2015	25/M	Incision and drainage	-	-
Tekkethil et al. [26]	2016	25/F	Antibiotics^a^, needle aspiration	NR	NR/NR
Wang et al. [14]	2013	23/M	Needle aspiration	-	-
Yang et al. [12]	2012	22/M	Needle aspiration	-	-
		54/M	Needle aspiration	-	-
		38/F	Needle aspiration	-	-

**Table 2:** Treatment of pediatric (≤18 years old) ITA cases ^a ^This patient reportedly had a tonsillectomy at a later date, but there is no evidence in the literature to confirm that the tonsillectomy occurred. NR - not reported; BID - two times a day; ITA - intratonsillar abscess

Author	Year	Age (years)/sex	Resolution treatment method	Antibiotic	Antibiotic dosage/duration (days)
Ben-Yaakov et al. [4]	2006	5/NR	Needle aspirations x2, IV antibiotics	ampicillin clavulanic acid	NR/NR
Childs et al. [3]	1991	16/F	Tonsillectomy	-	-
		16/M	Tonsillectomy	-	-
Hu et al. [27]	2022	17/F	Incision and drainage	-	-
Ormianer et al. [28]	2023	6/F	Needle aspiration, tonsillectomy	-	-
Ulualp et al. [13]	2013	5/F	IV antibiotics, incision and drainage	clindamycin ceftriaxone	NR/2
		13/F	IV antibiotics	clindamycin ceftriaxone	NR/5
		4/M	IV antibiotics	clindamycin	NR/2
		7/M	IV antibiotics	clindamycin	NR/4
		10/M	IV antibiotics	clindamycin ceftriaxone	NR/3
		18/M	IV antibiotics	clindamycin	NR/2
		15/F	IV antibiotics, incision and drainage	ampicillin sulbactam	NR/5
		13/F	IV antibiotics	clindamycin	NR/4
		16/M	IV antibiotics, tonsillectomy	clindamycin	NR/2
		8/F	IV antibiotics, tonsillectomy	clindamycin	NR/4
		4/M	IV antibiotics	clindamycin	NR/2
Wang et al. [14]	2013	12/M	IV antibiotics, tonsillectomy	ampicillin clavulanic acid	1200 mg BID/NR
		10/M	IV antibiotics, incision and drainage^a^	clindamycin	300 mg/NR
Yang et al. [12]	2012	11/F	Needle aspiration	-	-

**Table 3:** Treatment of ITA cases reported without a specific age or gender per individual patient ^a ^Full manuscript text is unavailable; only ITA case number and general treatment methods are included in the study abstract. ^b ^Method of antibiotic administration was not reported. NR - not reported; ITA - intratonsillar abscess

Author	Year	No of patients	Resolution treatment method	Antibiotic
Chen et al. [29]^a^	2014	10	Incision and drainage	-
Giurintano et al. [30]	2019	4	Incision and drainage	-
		18	Antibiotics^b^	NR

ITA typically involves a polymicrobial infection consisting of a combination of aerobic and anaerobic bacteria. Causative organisms often include Streptococcus pyogenes (group A Streptococcus), Enterococcus, Staphylococcus aureus, Haemophilus influenzae, Escherichia coli, Enterobacter, and Klebsiella. Unfortunately, the specific causative organism was not reported in the majority of reviewed studies, making it challenging to determine the exact incidence of each organism. As for the choice of antibiotic, a combination of broad-spectrum antibiotics is typically utilized, such as ampicillin, clavulanic acid, clindamycin, ceftriaxone, sulbactam, benzylpenicillin, metronidazole, and amoxicillin [12-16].

In our case, a simultaneous IV infusion of clindamycin and dexamethasone was administered to the patient. Clindamycin, with its broad spectrum of action against Staphylococci, Streptococci, Pneumococci, most anaerobic bacteria, Chlamydia trachomatis, and certain protozoa, has been previously reported to successfully clear ITA lesions. On the other hand, dexamethasone has proven effective in reducing pain and improving recovery time during PTA treatment [13,17-19].

A review of the literature and our patient's experience support a staged treatment approach as a future standard for ITA, with a first-line approach of IV antibiotics. If the infection persists, needle aspiration combined with IV antibiotics is recommended. If the abscess remains unresolved, I&D should be utilized. In cases where the ITA is refractory to I&D, tonsillectomy can be considered [5].

## Conclusions

This case report highlights the utilization of CT imaging and drainage with IV antibiotics for the diagnosis and management of acute ITA in an adult patient. By presenting this case and providing information from an extensive literature review, our objective is to enhance understanding about the symptoms and presentation of adult ITA to improve future diagnostic and treatment regimens.
